# Post‐Therapy Trajectories Following Brief Systemic Couple Therapy for Parents

**DOI:** 10.1111/famp.70114

**Published:** 2026-01-21

**Authors:** Joëlle Darwiche, Cindy Eira Nunes, Laura Vowels, Esther Liekmeier, Jean‐Philippe Antonietti

**Affiliations:** ^1^ Family and Development Research Center, University of Lausanne Lausanne Switzerland; ^2^ Psychological Sciences Research Institute, Catholic University of Louvain (UCLouvain) Leuven Belgium

**Keywords:** coparenting relationship, couple therapy, post‐therapy trajectories, romantic relationship

## Abstract

This study examined post‐therapy trajectories among parent couples who received either the Integrative Brief Systemic Intervention (IBSI)—targeting both romantic and coparenting relationships—or Brief Systemic Therapy as usual (BST‐as‐usual). Based on previous results showing comparable post‐treatment improvements across conditions, participants were analyzed together to identify the typical patterns of change couples follow after therapy. We assessed whether distinct trajectory groups could be identified over the 1‐year follow‐up and examined whether treatment‐related variables (therapy condition, number of sessions) and family characteristics (relationship duration, blended family status, number of children, age of youngest child) predicted group membership. Of the 101 Swiss randomized parent couples, 85 (44 IBSI, 41 BST‐as‐usual) provided data at post‐therapy, 6‐month, and 1‐year follow‐ups on individual symptomatology, romantic and coparenting relationship quality, and child adjustment. Mixed effects models first indicated that therapy gains were largely stable over time, with some parents reporting improvements in child adjustment, particularly men in BST‐as‐usual and women in IBSI. Using multiple factor analysis and hierarchical clustering (*n* = 72 couples), we identified five trajectories reflecting different configurations of individual distress and relational functioning. Multinomial logistic regression showed that both treatment characteristics and family context contributed to differentiating these pathways: IBSI was associated with more favorable trajectories, while having younger children or more children was linked to less optimal patterns. Clinically, identifying distinct post‐therapy trajectories underscores the importance of monitoring couples beyond treatment termination and tailoring support to those whose individual or family circumstances place them at higher risk of deterioration.

## 1 Introduction

Couple distress is of public health relevance, increasing the risk of psychological and physical health problems in partners (Gollan and Jacobson 2002). Couple therapy has been shown to be effective to support distressed partners in improving relationship quality and reducing individual symptomatology (Johnson and Lebow 2000; Shadish and Baldwin 2003). Meta‐analyses have reported medium (e.g., *d* = 0.585; Shadish and Baldwin 2005) to large (Hedges *g* = 0.91; Roddy et al. 2020) pre‐to‐post between‐groups effect sizes for relationship satisfaction. However, its impact on family dimensions such as coparenting or child adjustment has been far less studied, despite evidence that couple distress spills over into parenting behavior, affecting child adjustment (e.g., Zemp et al. 2018). Systems theory highlights the interdependence within families, suggesting that improving couple relationships through therapy can positively influence other subsystems, like the parent–child subsystem (Cox and Paley 1997). Despite Gollan and Jacobson's (2002) recommendations over 20 years ago to include them in research, outcomes such as coparenting and child adjustment remain underexplored in couple therapy studies.

Couple therapy for parents can focus on both romantic and coparenting relationships. Coparenting, which refers to the collaborative functioning between partners in their role as parents (McHale and Irace 2011), has been shown to exert a more immediate influence on children's socioemotional development compared to marital quality (e.g., Bonds and Gondoli 2007). In previous work, we examined the effectiveness of the Integrative Brief Systemic Intervention (IBSI; Darwiche et al. 2022), a six‐session therapy targeting both romantic and coparenting relationships, compared with Brief Systemic Therapy (BST‐as‐usual). Both interventions showed significant improvements in individual, romantic, coparenting, and family outcomes post‐treatment and at 6‐month follow‐up, with no major differences between them except that BST‐as‐usual couples sought more additional sessions. The similarity in outcomes may reflect shared systemic mechanisms of change across both interventions (Darwiche et al. 2023).

The objective of this study was to examine the 1‐year post therapy trajectories of parents who had participated in either IBSI or BST‐as‐usual. The follow‐up was extended to 12 months to trace the evolution of individual, couple, and family‐related outcomes. We examined not only overall trends of evolution in our sample but also the distinct patterns of change between couples—whether sustained, enhanced, or diminished. We further investigated whether treatment‐related variables (therapy condition, number of sessions) and family characteristics (relationship duration, blended family status, number of children in the household, and age of the target child) were associated with particular trajectories, thereby helping to explain why couples may follow different pathways after therapy.

### 1.1 Trajectories After Therapy Termination

Understanding how couples evolve after therapy is critical for several reasons. First, it helps establish whether treatment gains are maintained on average over time, indicating intervention effectiveness. Second, it sheds light on the diversity of relational pathways after therapy, since trajectories are often highly variable. Life events, for instance, may strain the relationship in the longer term, even when it was strengthened during therapy. Examining post‐therapy trajectories can therefore document these processes and clarify how couples adapt after therapy.

Research on the maintenance of gains indicates that couple therapy is effective (Lebow et al. 2012). However, sustaining these improvements remains challenging, with 35%–50% of couples experiencing declines or divorce post‐therapy (Snyder and Balderrama‐Durbin 2020). In their meta‐analysis of the marriage and family interventions literature, Shadish and Baldwin (2003) reported smaller follow‐up effect sizes (*d* = 0.52) compared to post‐treatment (*d* = 0.65), highlighting variability in sustained outcomes. For their part, Roddy et al. (2020) found in their meta‐analysis that participants sustained improved relationship satisfaction at long‐term follow‐ups (Hedge's *g* = 0.91).

Individual studies shed light on these dynamics. Christensen et al. (2006, 2010) found in a randomized trial comparing traditional and integrative behavioral couple therapy that two‐thirds of couples improved at 2 years post‐treatment despite initial decline in relationship satisfaction. By 5 years, improvements were sustained by half of the couples, with the remainder showing no change or deterioration. Non‐randomized studies also reported sustained benefits, including Emotionally Focused Couple Therapy (EFCT; Wiebe et al. 2017) and integrative approaches (Lundblad and Hansson 2006). In residential therapy, Tilden et al. (2010, 2020) observed initial gains, a 1‐year decline, and recovery at 3 years.

In summary, couple therapy effectively improves relationship satisfaction and interpersonal issues, but longitudinal studies reveal fluctuating trajectories with periods of decline and recovery, highlighting the dynamic nature of outcomes. Documenting these post‐therapy trajectories is therefore important for understanding the long‐term change processes and guiding clinical practice (Lebow and Snyder 2022).

### 1.2 Factors Associated With Post‐Therapy Trajectories

A key challenge in couple psychotherapy research is to move beyond immediate outcomes and identify affect long‐term effectiveness. Understanding why some couples sustain gains while others relapse is relevant for clarifying post‐therapy trajectories and guiding tailored interventions (Doss et al. 2019). However, common prognostic indicators, such as demographic and intrapersonal factors (e.g., age, education, marriage length, or initial relationship quality), have generally provided little explanatory power in predicting long‐term outcomes (Atkins et al. 2005; Doss et al. 2019). While some studies identified communication dynamics and emotional arousal as predictors of outcomes (Baucom et al. 2009), others highlighted commitment‐related factors, such as longer marital duration and fewer steps toward divorce, as stronger indicators of marital stability at 5 years (Baucom et al. 2011). More recent work points to changes in attachment processes (Wiebe et al. 2017) and baseline distress levels (Roddy et al. 2020) as meaningful predictors, but overall findings remain mixed, underscoring the heterogeneity of couples' trajectories beyond therapy.

Among treatment‐related variables potentially associated with post‐therapy trajectories, the therapy condition is typically expected to favor more positive outcomes compared to control or alternative conditions, though findings are nuanced. For example, Integrative Behavioral Couple Therapy (IBCT) produced significantly greater improvements than Traditional Behavioral Couple Therapy up to the 2‐year follow‐up (Baucom et al. 2009), but no differences were observed at 5 years (Baucom et al. 2011). The number of sessions has also been highlighted, with evidence of a curvilinear dose–response association, underscoring the need for follow‐up data to assess the long‐term effects (Robinson et al. 2019).

Family and contextual characteristics may also be linked to post‐treatment trajectories: longer marital duration has been linked to more favorable outcomes (Baucom et al. 2009), whereas blended family status (Teachman 2008), having more children, or parenting younger children may increase relational strain and risk of instability (Fang et al. 2024). Coparenting complexity and stress associated with certain developmental stages (e.g., early childhood or adolescence) may plausibly undermine the sustainability of therapeutic gains.

In conclusion, more research is needed to examine how treatment‐related variables and family characteristics are associated with the diverse trajectories couples may follow after therapy. Identifying such associations is important for clarifying the contexts in which couples are more likely to sustain therapeutic gains, experience fluctuations, or face declines over time. A better understanding of these factors could ultimately inform the development of more tailored interventions and post‐therapy support strategies aimed at fostering longer term relationship stability and well‐being.

### 1.3 Main Objectives and Hypotheses

This study examined post‐therapy trajectories of couples who had participated in either IBSI or BST‐as‐usual (Darwiche et al. 2023). These trajectories were constructed using data from three post‐treatment assessments—immediately after therapy, at 6 months, and at 1‐year—covering parents' individual symptomatology, the quality of their romantic and coparenting relationships, and parent‐reported child adjustment.

We hypothesized that therapy‐related improvements would generally be maintained at the one‐year follow‐up (Research Question 1). However, we expected distinct post‐therapy trajectories to emerge (Research Question 2), with some couples showing further improvement or stability, and others experiencing deterioration, depending on individual and relational resources for maintaining change. We further expected both treatment (therapy condition, number of sessions) and family‐related factors (relationship duration, family type—blended vs. non‐blended, number of children, and age of the youngest child) to be associated with trajectory group membership (Research Question 3). Specifically, we anticipated that parents in the IBSI condition would be more likely to follow positive trajectories, as this intervention targeted both the romantic relationship and the coparenting alliance, thereby strengthening two key relational domains important for long‐term couple functioning. In contrast, we expected more sessions to relate to less optimal trajectories, given the model's brief format. Finally, longer relationship duration was expected to predict more favorable outcomes, whereas we anticipated family characteristics such as blended family status, having more children, or parenting younger children to be associated with less optimal trajectories.

This study contributes valuable data on post‐therapy trajectories after systemic couple therapy for parents, across individual, couple, and family‐related outcomes. Unlike many existing trials which focus on US‐based models like IBCT and EFCT, this research examines a systemic treatment model in European/Swiss settings under clinically representative conditions. Although systemic therapy is widely practiced in many European contexts and supported by long‐standing clinical traditions, empirical evidence from research in real‐world settings remains scarce and is particularly limited regarding the trajectories that couples follow after therapy.

## 2 Method

Below we provide a summary of the methodology for the study. Full details can be found in Darwiche et al. (2023).

### 2.1 Participants and Procedure

Couples were recruited from consultation centers in French‐speaking Switzerland. At recruitment, couples were informed that the study examined the effects of couple therapy for parents, addressing both romantic and coparenting relationships while focusing on their presenting concerns. They were informed that they would be randomly assigned to either IBSI, with a stronger focus on both romantic and coparenting relationships, or to BST‐as‐usual. Inclusion criteria required being parents of at least one child aged 0–16 years and engaged in a couple relationship (married or not). Exclusion criteria were situations of violence, maltreatment, or abuse preventing psychotherapeutic treatment, as well as the current severe psychiatric disorder of one or both partners. Of the 447 couples approached, 308 declined (69%), whereas 38 others had initially accepted but were ultimately not included for reasons unrelated to refusal (e.g., pilot cases or couples who did not start therapy).

Eligible couples were randomly assigned to IBSI (*n* = 51) or BST‐as‐usual (*n* = 50) using block randomization. Participants were compensated at each assessment ($100 post‐therapy, $100 at follow‐up 1, $200 at follow‐up 2). The study included 33 systemic psychotherapists, 19 of whom were trained in IBSI through workshops, supervised therapy, and clinical practice, with monthly supervision during the study. Both IBSI and BST‐as‐usual therapists had comparable levels of experience and systemic programs completion rates, with no significant differences observed between the groups. The study was approved by the Ethics Committee of University of Lausanne, Switzerland.

Analyses were conducted with couples who completed post‐treatment assessments (*N* = 85; IBSI = 44, BST‐as‐usual = 41), with participant demographics reported in Table 1. Participation declined slightly at follow‐up, with 76 couples providing data at 6 months (IBSI = 41, BST‐as‐usual = 35) and 72 couples at 1‐year (IBSI = 38, BST‐as‐usual = 34). No significant demographic differences were found between the two treatment groups, except that men in the IBSI group were older than those in the BST‐as‐usual group, *t*(94) = −2.73, *p* = 0.008.

**TABLE 1:** Demographic characteristics of the participants.

	Women	Men
Age, *M* (SD)	37.8 (7.37)	39.83 (8.08)
Nationality, *n* (%)		
Swiss	63 (75)	65 (77.4)
European	16 (19)	16 (19.1)
Other	5 (6)	3 (3.6)
Highest educational level, *n* (%)		
Elementary School	7 (8.3)	9 (10.7)
Vocational School	14 (16.7)	25 (29.8)
High School	8 (9.5)	8 (9.5)
University	42 (50)	31 (36.9)
Other	13 (15.5)	11 (13.1)
Marital status, *n* (%)		
Married/Common‐law	61 (72.6)	
Cohabiting	11 (13.1)	
Separated/Divorced	12 (14.3)	
Blended families, *n* (%)	13 (15.5)	
Duration of relationship (years), *M* (SD)	12 (7.22)	
Duration of difficulties, *M* (SD)	3.65 (3.61)	2.98 (2.64)
Area of main issues, *n* (%)		
Marital issues	61 (72.6)	59 (70.2)
Coparenting issues	14 (16.7)	20 (23.8)
Marital and coparenting issues	8 (9.5)	3 (3.6)
No response	1 (1.2)	2 (2.4)
Number of cohabiting children, *M* (SD)	1.98 (1.66)	
Age of youngest child, *M* (SD)	5.66 (4.63)	

*Note:* Socio‐demographic data were missing for one couple.

Abbreviations: *M*, mean; SD, standard deviation.

Comparisons of post‐test scores showed that women and men who dropped out before the 1‐year follow‐up reported significantly lower romantic satisfaction scores immediately after therapy, *t*(18.14) = 2.91, *p* = 0.009. In addition, women who dropped out reported lower perceived coparenting support, *t*(18.32) = 2.46, *p* = 0.024.

At each post‐treatment assessment, couples completed measures of individual distress (i.e., individual symptomatology and depression), romantic relationship quality, coparenting relationship quality (i.e., support and conflict), and child adjustment. Given the proportion of missing data (33%, calculated across all variables and the three measurement points), we first verified that data were missing completely at random using Little's test, *χ* ^*2*^(229) = 249, *p* = 0.176. Accordingly, we retained the full set of available data (*N* = 85) without excluding any couple or individual, except for analyses examining the distinct patterns of trajectories between couples (see Section 2.5), which were conducted with a subsample of 72 couples.

### 2.2 Integrative Brief Systemic Intervention (IBSI)

IBSI is a manualized brief systemic couple intervention designed for parents seeking couple therapy (see Darwiche et al. 2022 for more details). It addresses the couple's presenting problem, considering its specific impact on the romantic and coparenting domains. The intervention consists of about six sessions over 6 months (*M* = 5.83, SD = 0.59) divided into three phases. The first session establishes a therapeutic space and sets shared objectives. Sessions 2–5 focus on achieving these goals using coparenting research and systemic therapy techniques. The final session reviews progress and addresses relapse prevention.

### 2.3 Brief Systemic Therapy‐As‐Usual (BST‐As‐Usual)

BST‐as‐usual is a non‐manualized systemic intervention widely used in the French‐speaking Swiss community as couple therapy. Like IBSI, it involves approximately six sessions over 6 months (*M* = 5.98, SD = 0.84). BST‐as‐usual focuses on improving the romantic relationship through techniques that foster positive behaviors, enhance communication, and build problem‐solving skills. It incorporates concepts and techniques from various schools of systemic therapy (e.g., de Shazer 1985; Haley 1973; Minuchin 1974).

### 2.4 Measures

#### 2.4.1 Individual Symptomatology

Individual symptomatology was assessed with the Outcome Questionnaire (OQ‐10.2; Lambert et al. 2000), a 10‐item scale (scores: 0–40; higher scores indicate greater symptomatology). Across the assessment points, reliability ranged from *α* = 0.84–0.92 (women) and α = 0.76–0.88 (men).

#### 2.4.2 Depression

Depression was measured using the Patient Health Questionnaire (PHQ‐9; Spitzer et al. 1999), a 9‐item scale (scores: 0–27; higher scores indicate more severe depression). Across the assessment points, reliability was *α* = 0.84–0.85 (women) and *α* = 0.82–0.90 (men).

#### 2.4.3 Romantic Relationship Quality

Romantic Relationship Quality was evaluated with the Dyadic Adjustment Scale (DAS; Spanier 1976), a 32‐item scale (scores: 0–151; higher scores reflect better relationship quality). Across the assessment points, reliability ranged from *α* = 0.92–0.94 (women) and *α* = 0.89–0.95 (men).

#### 2.4.4 Coparenting Relationship Quality

Coparenting Relationship Quality was assessed along two dimensions. *Coparenting Support* was assessed using the Parenting Alliance Measure (PAM; Konold and Abidin 2001), a 20‐item scale (scores: 20–100; higher scores indicate stronger support). Across the assessment points, reliability ranged from *α* = 0.94–0.96 (women) and *α* = 0.95–0.96 (men). *Coparenting Conflict* was measured with the Coparenting Inventory for Parents and Adolescents (CI‐PA; Teubert and Pinquart 2011; Zimmermann et al. 2020), a 16‐item scale (scores: 0–5; higher scores indicate greater conflict and triangulation). Across the assessment points, reliability ranged from *α* = 0.85–0.90 (women) and *α* = 0.87–0.89 (men).

#### 2.4.5 Child Adjustment

Child adjustment was measured via parent reports in the Strengths and Difficulties Questionnaire (SDQ; Goodman 1997), a 20‐item scale (scores: 0–40; higher scores reflect more difficulties), with both parents independently completing the measure. Across the assessment points, reliability ranged from *α* = 0.76–0.87 (women) and *α* = 0.76–0.83 (men).

### 2.5 Data Analysis

To determine whether individual symptomatology and depression, romantic relationship quality, coparenting relationship quality, and child adjustment could be considered stable from post‐test to follow‐up 1 and follow‐up 2, we used mixed effects models with individuals nested within dyads (Raudenbush and Bryk 2010). The analysis followed three steps: first, an intercept‐only model was specified, assuming that the level of the variable of interest remained constant across post‐test, follow‐up 1, and follow‐up 2, for women and men in both the IBSI and BST‐as‐usual groups. In a second step, a more complex model was specified, allowing the variable to follow a linear trajectory over time, with different slopes for women and men in each group. In a third step, the intercept‐only model (simplified) was compared with the linear trajectory model (complex) using a likelihood ratio test. The null hypothesis indicated stability, while the alternative hypothesis suggested significant linear change for at least some participants. These analyses, conducted separately for each of the six outcome variables, were performed using R (R Core Team 2024) with the package nlme (Pinheiro et al. 2022).

Next, we created groups of couples (combining both conditions) using men's and women's scores on all six outcome variables measured at post‐test, follow‐up 1, and follow‐up 2 (18 variables for each gender). For this set of analyses, given the proportion of data missing completely at random, we decided to exclude couples for whom more than half of the data across all variables and time points were missing (*n* = 13), using thus a subsample of 72 couples. The remaining missing data were handled using single imputation with the mice package (van Buuren and Groothuis‐Oudshoorn 2011) in R.

Multiple factor analysis (MFA; Abdi et al. 2013; Husson et al. 2017) was applied initially to reduce data dimensionality. MFA, a weighted Principal Component Analysis, is suitable when individuals or dyads are described by several groups of variables, as it balances the influence of each group of variables in the analysis (Husson et al. 2010). MFA may not be the conventional choice for modeling the temporal evolution of individuals or groups; it was adopted as it enables the simultaneous representation of repeated observations across time and the identification of overarching trends, without the need to explicitly model individual trajectories. This approach translates continuous, interval‐level data into profiles that may inform therapeutic understanding and future research. To test the robustness, we also conducted latent class growth analyses and compared them with MFA; results (see Table S1) showed substantial overlap between latent classes and the cluster solution, supporting the validity of the identified trajectories (see also Dazy et al. 1996; Millsap and Meredith 1988).

Based on the results of the MFA, we reduced the space with three dimensions retained which were then fed into Hierarchical Clustering on Principal Components analysis (HCPC; Husson et al. 2010) to create clusters. The Krzanowski‐Lai (KL) criterion (Krzanowski and Lai 1988) was used to determine the optimal number of clusters by identifying the point where adding more clusters no longer significantly improved clustering quality. It evaluates both cohesion within clusters and separation between clusters to achieve this balance. We chose five groups to be retained for the HCPC according to the KL criterion. These groups were analyzed to explore differences in the outcome variables from which they were constructed, using the FactoMineR (Lê et al. 2008) and NbClust packages (Charrad et al. 2014) in R.

Finally, we tested whether several socio‐demographic variables predicted group assignment. We conducted multinomial logistic regression with several predictors: the therapy condition, number of sessions, duration of the couple's relationship, type of family (blended family or not), number of children living with the couple, and age of the youngest child (target child). Analyses were conducted using the nnet package (Venables and Ripley 2002) in R.

## 3 Results

We assessed post‐therapy trajectories in three stages. First, we examined whether the improvements achieved during therapy were maintained across the follow‐up period. Second, we investigated whether couples differed in their post‐therapy trajectories over the year. Finally, we explored whether, and which, treatment‐related variables and family characteristics predicted membership in specific trajectory groups.

### 3.1 Stability of Therapy Gains up to the One‐Year Follow‐Up

The results from the mixed effects models showed that the intercept only model (stability, including only the intercepts for each group for men and women separately) was no worse significantly compared to the full model (also including an interaction between group and time) which included time on five of the six outcome variables (see Table S2 for details regarding number of observations, empirical means, standard deviations of target variables; see Table 2 for the model comparisons; see Tables S3–S7 for full results of the full models). Therefore, we retained the simpler model for five of the outcomes which suggested that the gains made in therapy stayed stable over time for individual symptomatology, depression, romantic relationship quality, coparenting support, and coparenting conflict. The only outcome variable that changed over time was the parents' reports of child adjustment (see Table 3 for the results). More specifically, the results showed that men in the BST group and women in the IBSI group perceived their children as doing better over time suggesting that children continued to experience fewer difficulties over time up to 1 year after therapy. As part of the peer‐review process, we conducted supplementary analyses on mean differences between post‐test on the one hand, and the two follow‐ups, on the other hand. Results indicated improved perceptions of child adjustment for both parents and reduced coparenting conflict for men, with linear mixed models confirming these patterns across 6‐month and 1‐year follow‐ups (see Tables S8 and S9).

**TABLE 2:** Results of the likelihood ratio test comparing the stability and change models.

	AIC	BIC	Loglik	L.ratio	*p*
OQ null model	2864.10	2913.86	−1420.05		
OQ with time	2868.20	2934.54	−1418.10	3.90	0.419
PHQ null model	2618.33	2668.08	−1297.16		
PHQ with time	2621.92	2688.26	−1294.96	4.41	0.353
DAS null model	3656.40	3705.50	−1816.20		
DAS with time	3661.56	3727.02	−1814.78	2.85	0.584
PAM null model	3421.84	3471.57	−1698.92		
PAM with time	3421.11	3487.41	1694.55	8.74	0.068
CIPA null model	615.54	665.24	−295.77		
CIPA with time	617.60	683.87	−292.80	5.94	0.204
SDQ null model	2482.83	2530.75	−1229.41		
SDQ with time	2476.45	2540.36	1222.23	14.37	**0.006**

*Note:* Bold values indicates statistically significant.

Abbreviations: CI‐PA, Coparenting Inventory for Parents and Adolescents; DAS, Dyadic Adjustment Scale; OQ, Outcome Questionnaire; PAM, Parenting Alliance Measure; PHQ, Patient Health Questionnaire; SDQ, Strengths and Difficulties Questionnaire.

**TABLE 3:** Results of the change model for parent‐reported child adjustment.

Predictors	SDQ		
	Estimates	CI	*p*
G0 * M	11.37	9.51 to 13.23	**< 0.001**
G0 * W	11.32	9.29 to 13.35	**< 0.001**
G1 * M	9.74	7.99 to 11.50	**< 0.001**
G1 * W	10.34	8.47 to 12.22	**< 0.001**
G0 * M * time	−1.04	−2.00 to −0.08	**0.036**
G0 * W * time	−0.93	−1.90 to 0.04	0.064
G1 * M * time	−0.54	−1.43 to 0.35	0.239
G1 * W * time	−1.24	−2.12 to −0.37	**0.006**
**Random effects**			
*σ* ^2^	14.62		
*τ*_00 Couple_	26.39		
*τ*_11 Couple.M_	29.91		
*τ*_11 Couple.W_	20.33		
*ρ*_01_	−0.64		
	−0.43		
ICC	0.64		
*N*_Couple_	82		
Observations	401		
Marginal *R* ^2^/Conditional *R* ^2^	0.023/0.652		

*Note:* G0, BST‐as‐usual; G1, IBSI; M, man; W, woman. Bold values indicates statistically significant.

### 3.2 Different Trajectories After Therapy

The HCPC results showed five distinct groups of couples based on their mean levels of the outcome variables at all three post‐treatment assessments (i.e., post‐test, follow‐up 1, and follow‐up 2; see Table 4 for the results). The first two groups both showed stable outcomes following therapy, with both men and women reporting similar and stable, relatively high scores over time. Group 2 (*n* = 27) had somewhat lower scores compared to Group 1 (*n* = 18) across the trajectory measured at post‐therapy, follow‐up 1, and follow‐up 2. Group 3 (*n* = 7) had higher individual symptomatology and depression scores in men compared to their partners, whereas Group 4 (*n* = 10) showed higher individual symptomatology and depression scores in women. Women in Group 4 also followed a trajectory of lower romantic relationship quality and coparenting relationship quality than their partners. Finally, both men and women in Group 5 (*n* = 10) showed a consistent trajectory of lower romantic relationship quality and coparenting relationship quality but not across individual symptomatology or child adjustment. The results indicate that couples' trajectories following therapy can be grouped into distinct clusters: Groups 3 and 4 are defined by higher levels of individual distress (i.e., individual symptomatology and depression) in one partner, while Group 5 is characterized by low relationship quality in both partners.

**TABLE 4:** Summary of the five post‐therapy trajectories.

Group	*N*	Overall description	Key outcomes
1	18	Stable high‐functioning couples	Sustained positive outcomes after therapy
2	27	Stable moderate‐functioning couples	Stable outcomes at a moderately lower level
3	7	Elevated individual distress in men	Partner asymmetry: male‐specific vulnerability post‐therapy
4	10	Elevated individual distress in women	Partner asymmetry: female‐specific vulnerability post‐therapy
5	10	Low relationship quality in both partners	Chronic relational difficulties despite no marked individual distress

To illustrate the above results, Figures S1–S6 show the distinct trajectories in the outcome variables for each of the five groups. To help characterize post‐therapy trajectories in relation to pre‐therapy scores, these figures included the evolution of scores from pre‐ to post‐therapy in dashed lines; however, pre‐therapy scores were not taken into account when creating the five groups.

### 3.3 Factors Associated With Trajectory Group Membership

Based on the multinomial logistic regression, we were able to predict the odds of being assigned to Groups 2 and 4 against Group 1. Specifically, couples in the IBSI condition were more likely to belong to Group 1—characterized by a stable trajectory with high romantic and coparenting relationship quality and low individual symptomatology and depression—than to Group 2, which also showed stability but with relatively lower scores, OR = 0.12, 95% CI [0.02; 0.68], *p* = 0.017. Moreover, couples with younger children were more likely to be included in Group 2, OR = 0.70, 95% CI [0.52; 0.95], *p* = 0.020, compared with Group 1. Couples with a longer relationship duration were more likely to belong to Group 4—characterized by women reporting higher individual symptomatology and depression than their partners, together with lower romantic relationship quality and coparenting quality—compared with Group 1, OR = 1.54, 95% CI [1.07; 2.22], *p* = 0.019. Some predictions were also marginally significant, as couples with more children living at home, OR = 3.17, 95% CI [1.00; 10.10], *p* = 0.051, tended to more often belong to Group 4 than Group 1. No other predictors were significant (i.e., number of sessions, family type–blended vs. non‐blended), and no significant predictions were found for odds of belonging to Group 3—characterized by higher individual distress in men—or to Group 5, marked by low romantic relationship satisfaction and coparenting relationship quality but relatively unaffected individual distress and child adjustment, compared with Group 1.

## 4 Discussion

This study aimed to examine post‐therapy trajectories among couples who participated in either IBSI or BST‐as‐usual over the year following treatment termination. Trajectories were derived from outcomes assessing various dimensions of couple and family dynamics, extending beyond the traditional focus on romantic relationship satisfaction and individual distress to also include parents' perceptions of coparenting quality (support and conflict) and child's adjustment.

First, the findings confirmed that improvements in individual distress, romantic relationship quality, and coparenting support and conflict observed 6 months after therapy (Darwiche et al. 2023) were maintained at the 1‐year follow‐up across both treatment groups. Notably, parents' perceptions of their child's adjustment improved during this period, particularly for men in the BST‐as‐usual group and women in the IBSI group, suggesting a delayed positive impact of therapy on children through improved parental well‐being. However, as these results were not significant for all parents, further research is needed to better understand the link between couple therapy and child outcomes.

Sustaining long‐term gains in couple therapy remains a critical challenge. While therapy reliably improves relationship satisfaction, only about 40% of couples achieve satisfaction levels comparable to non‐clinical populations (Lebow et al. 2012), and outcomes vary: some couples continue to improve, others remain distressed, deteriorate, or separate. New vulnerabilities and stressors, such as the loss of loved ones or transitioning from a family‐focused to a couple‐focused life after their children have grown, can hinder the application of previously learned coping strategies. This study contributes to the literature on systemic treatments by indicating that such interventions may foster parent couples' ability to anticipate and cope with future challenges. Achieving enduring change in relationships over the long‐term (5 years or more) can be challenging through couple therapy alone, with a key outcome being the development of the couple's ability to seek professional support during vulnerable periods.

Overall, the findings suggest that therapeutic gains were maintained at 1‐year follow‐up across both treatment groups. However, such global results may conceal important heterogeneity, leading this study to examine couples' post‐therapy trajectories and identify distinct patterns of change over the year following treatment termination. Five groups emerged based on individual and relationship outcomes, revealing five distinct post‐therapy trajectories characterized by varying levels of individual symptomatology (i.e., individual symptoms and depression) and relationship quality (i.e., romantic and coparenting relationships). Groups 1 and 2 exhibited stable trajectories with high relationship quality and low symptomatology, though Group 2 scored slightly lower than Group 1. In contrast, Groups 3 and 4 revealed gender‐specific patterns of individual distress: men in Group 3 reported higher individual distress, while women in Group 4 reported both greater individual distress and lower romantic and coparenting relationship quality. Group 5, meanwhile, was characterized by low romantic and coparenting relationship quality, although individual distress and child adjustment were less affected. These observations point to individual distress (Groups 3 and 4) and relationship quality (Group 5) as underlying dimensions structuring the diverse trajectories couples follow after therapy.

Groups 1 and 2 representing the majority (45 out of 72 couples) maintained stable, high levels of relationship quality and low levels of individual symptomatology after therapy, demonstrating the intervention's overall effectiveness. In contrast, Groups 3 and 4 showed gender‐specific imbalances, with one partner reporting lower satisfaction across several domains of couple and family life. According to the weak link hypothesis (Park et al. 2023), the more vulnerable partner may undermine overall relationship quality, increasing the risk of long‐term deterioration. Conversely, the strong link hypothesis (Schoebi et al. 2012) suggests that the healthier partner can buffer the relationship by assuming greater responsibility for its stability, but this protective role may also entail emotional costs over time. In cases where one partner's vulnerability reflects individual psychopathology, couple‐based interventions for psychopathology (Baucom et al. 2014) may provide a promising way to leverage the relationship as a therapeutic resource while preventing additional burden on the healthier partner. Group 5 couples (10/72) showed declines in romantic and coparenting relationship quality, with the degradation in romantic relationship quality potentially signaling a heightened vulnerability to separation or divorce. The decline in coparenting relationship quality is particularly concerning, as a strong coparenting relationship is essential for making joint decisions in the children's best interests, even after separation (Ahrons 2007). Research shows that high‐quality coparenting facilitates shared custody arrangements, which are associated with better child outcomes compared to sole custody (Vowels et al. 2023). Couple therapy that includes work on coparenting can help couples manage separation while preserving their parenting bond, ultimately benefiting the children (Darwiche et al. 2022).

Finally, when considering treatment‐related variables, couples in the IBSI condition were more frequently found in the most favorable group (Group 1: stable high satisfaction and well‐being) compared with Group 2, which was also stable but characterized by lower satisfaction and well‐being. This suggests that interventions explicitly targeting both romantic and coparenting dimensions may provide a stronger foundation for sustaining therapeutic gains. At the same time, post‐therapy improvements were maintained up to 1 year across both IBSI and BST‐as‐usual, with no significant overall differences between conditions, which nuances this interpretation.

Family context also appeared to be associated with couples' post‐therapy trajectories. Couples with younger children were more frequently in Group 2 than in Group 1, suggesting that the early parenting period may introduce challenges that can attenuate the benefits of therapy. Similarly, the marginal association with a higher number of children at home (more often linked to Group 4, where women reported greater difficulties) resonates with existing evidence on the strain that family load places on partners—especially mothers (Ren et al. 2024)—and suggests that these burdens may hinder the consolidation of therapy gains.

Longer relationship duration was also linked to Group 4, characterized by women's higher symptomatology and lower romantic and coparenting satisfaction. It may indicate that women—who are often the ones initiating therapy—can be caught in entrenched relational patterns that are harder to shift in long‐standing relationships, or that gendered burdens in long‐term partnerships may be difficult to address within the scope of brief couple therapy (Sutherland et al. 2017).

Taken together, these findings suggest that, beyond treatment condition, the family life‐cycle stage and relationship characteristics are important to consider when seeking to understand how couples maintain or struggle with therapy gains. The absence of significant associations for some trajectories (Groups 3 and 5) further indicates that other interpersonal or contextual processes, not assessed in this study, may be critical in explaining why certain couples remain vulnerable after therapy.

### 4.1 Clinical Implications

The present findings highlight the value of examining post‐therapy trajectories rather than relying solely on average treatment effects. The identification of distinct pathways—ranging from stable improvements to persistent vulnerabilities or gender‐specific imbalances—shows that therapy outcomes are not uniform. Recognizing such heterogeneity is essential for tailoring support to couples' needs, for example through booster sessions, on‐demand follow‐up, or targeted interventions when one partner remains particularly vulnerable (e.g., mothers facing a heavy parental mental load). Beyond treatment modality, family characteristics such as parenting young children or managing heavier family responsibilities may also require additional resources to consolidate therapeutic gains. Viewing the post‐therapy period as a critical phase in its own right can help clinicians anticipate life transitions and stressors that may undermine prior improvements and provide timely support to sustain relationship stability and family well‐being over the long‐term.

### 4.2 Limitations and Future Directions

While this study provides valuable insights, findings should be interpreted with caution given the small sample size and the modest acceptance rate (one third of eligible couples). Future research should replicate findings with larger and more diverse populations to confirm and compare trends across groups, while also ensuring greater feasibility to enhance recruitment rates. Moreover, as several trajectories appeared non‐linear, future research should consider alternative modeling approaches (e.g., quadratic or spline) to better capture possible curvilinear patterns over time. Lastly, more frequent follow‐up assessments could clarify long‐term change patterns to determine whether the changes observed after 1‐year reflect a sustained negative trend or a trajectory characterized by fluctuations influenced by life events and periods of vulnerability.

## 5 Conclusion

This study advances the systemic literature on couple therapy for parents by demonstrating its potential to foster long‐term improvements in family‐related domains, including coparenting satisfaction and child adjustment. By identifying five distinct trajectories, our findings underscore that therapeutic outcomes are not uniform: while some couples maintain positive changes, others face persistent vulnerabilities or gender‐specific difficulties. Future research should clarify the conditions under which therapy gains are sustained by considering relational and contextual factors such as, for example, power dynamics, parental mental load, or socio‐economic stressors. Broadening the evidence base to include diverse family constellations—such as interracial couples, same‐gender parents (Kousteni and Anagnostopoulos 2020), and families from varied socio‐economic backgrounds—will be key to ensuring that systemic couple therapy remains a flexible and inclusive intervention, responsive to the realities of contemporary couple and family life.

## Funding

This work was supported by Schweizerischer Nationalfonds zur Förderung der Wissenschaftlichen Forschung, Grant SNF 159437.

## Conflicts of Interest

The authors declare no conflicts of interest.

## Supporting information


**Data S1:** famp70114‐sup‐0001‐supinfo.docx.

## Data Availability

The data that support the findings of this study are available on request from the corresponding author. The data are not publicly available due to privacy or ethical restrictions.
