# Supplementary material for: Post‐Therapy Trajectories Following Brief Systemic Couple Therapy for Parents
Source: Fam Process. 2026 Jan 21;65(1):e70114. doi: 10.1111/famp.70114 (PMC12824436; doi:10.1111/famp.70114)
Supplement: Supplementary file 1 — Data S1: famp70114‐sup‐0001‐supinfo.docx. [file FAMP-65-0-s001.docx]

**Table S1**

*Comparison of Latent Class Growth Analyses and A Cluster Solution Based on Hierarchical Clustering (N = 5)*

| **Dependent variable for the classes** | Best solution (with fit) | Model description | Association between latent class growth solution and hierarchical solution |
| --- | --- | --- | --- |
| Women’s individual symptomatology | 2 classes  With no time in mixture  BIC = 1479.1 | Class 1(69%) M = 10.60, SE = 0.59, p < .001  Class 2 (31%) M= 19.96, SE = 0.77, p < .001  No significant changes over time (post-fu1 p = .755; post-fu2 p = .148)  Class 2 women are less likely to be in Class 1 [OR = 0.69, SE = 0.28, p = .012] | G(4) = 22.6, p <.001  Cramer's V = .57 |
| Men’s individual symptomatology | 3 classes  With no time in mixture  BIC = 1440.6 | Class 1(30.7%) M = 8.23, SE = 0.87, p < .001  Class 2(54.5%) M = 14.33, SE = 0.74, p < .001  Class 3(14.8%) M = 23.35, SE = 0.86, p < .001  No significant changes over time (post-fu1 p = .245; post-fu2 p = .269)  Class 3 men are less likely to be attributed to Class 2 [OR = 1.30, SE = .36, p < .001] | G(8) = 45.5, p < .001  Cramer's V = .62 |
| Women’s romantic satisfaction | 3 classes  With no time in mixture  BIC: 1469.6 | Class 1 M = 111.33, SE= 2.07, p < .001  Class 2 M = 91.26, SE = 2.29, p < .001  Class 3 M = 56.87, SE = 3.57, p < .001  No significant changes over time (post-fu1: p = .503; post-fu2: p = .407)  Class 3 women are significantly less likely than to be in Class 2 [with Class 2: OR = -1.36, SE = 0.46, p = .003] | G(8) = 26.9, p < .001  Cramer's V: .59 |
| Men’s romantic satisfaction | 3 classes  With no time in mixture  BIC: 1386.9 | Class 1 15.6%; Class 2 50%; Class 3 34.4%  Class 1 M= 124.46, SE = 2.73, p < .001  Class 2 M = 108.08, SE= 1.72, p < .001  Class 3 M = 83.85, SE = 1.82, p < .001  No changes over time (post-fu1 p = .094; post-fu2 p = .535) | G(8) = 32.6, p < .001  Cramer's V: .56 |
| Women’s coparenting support | 3 classes  With no time in mixture  BIC = 1778.9 | Class 1(19.5%) M = 94.76, SE = 1.56, p < .001  Class 2 (65.5%) M = 78.00, SE = 1.21, p < .001  Class 3 (14.9%) M = 58.18, SE = 1.95, p < .001  No significant change over time (post-fu1 p = .403, post-fu2 p= .449)  Class 3 women reported significantly less coparenting support than Class 2 women [OR = 1.42, SE = 0.36, p < .001] | G(8) = 52.0, p < .001  Cramer's V: .65 |
| Men’s coparenting support | 3 classes  With no time in mixture  BIC = 1724.0 | Class 1(40.2%) M = 92.07, SE = 1.21, p < .001  Class 2(48.3%) M = 78.15, SE = 1.51, p < .001  Class 3(11.5%) M = 62.41, SE = 2.38, p < .001  No significant changes over time (post-fu1 p = .304; post-fu2 p = .080)  Class 3 men are significantly less likely to be attributed to Class 1 [OR = 1.33, SE = 0.49, p = .006] and Class 2 [OR = 1.33, SE = 0.48, p = .005] | G(8) = 59.7, p < .001  Cramer's V: .78 |
| Women’s coparenting conflict | 3 classes  With no time in mixture  BIC = 345.5 | Class 1(41.4%) M = 0.53, SE = 0.06, p < .001  Class 2(47.1%) M = 1.30, SE = 0.06, p < .001  Class 3(11.5%) M = 2.22, SE = 0.10, p < .001  No changes over time  (post-fu1 p = .126, post-fu2 p= .901)  Class 3 less likely to be in Class 2[with Class 2: OR = -1.40, SE = .42, p < .001] | G(8) = 39.9, p < .001  Cramer's V: .52 |
| Men’s coparenting conflict | 3 classes  With no time in mixture  BIC = 1386.9 | Class 1(64.0%) M = 0.62, SE = 0.04, p < .001  Class 2 (24.4%) M = 1.45, SE = 0.07, p < .001  Class 3(11.6%) M = 2.15, SE = 0.10, p < .001  Significant increase of coparenting conflict from post to fu2 (B = 0.11, SE = 0.06, p = .048)  Class 3 are significantly less likely to be attributed to Class 1 [OR = 1.73, SE = 0.42, p < .001] | G(8) = 42.3, p < .001  Cramer's V: .57 |
| Child difficulties perceived by women | 3 classes  With time in mixture  BIC = 1318.1 | Class 1(54.9%) M = 5.41, SE = 0.78, p < .001 with no significant changes over time (post-fu1 p = .987; post-fu2 p = 854)  Class 2(9.8%) M = 14.65, SE = 1.20, p < .001 with no significant changes from post to fu1 (p = .072) but significant decreases from post to fu2 [B = -3.74, SE = 1.39, p = .007]  Class 3(35.4%) M = 26.5, SE = 1.66, p < .001 with significant decreases over time (post-fu1 B = -12.5, SE = 2.28, p < .001; post-fu2 B = -5.22, SE = 2.33, p = .025]  Class 3 women are significantly less likely to be attributed to Class 1 [OR = -1.58, SE = 0.42, p < .001] | G(8) = 28.4, p < .001  Cramer's V: .52 |
| Child difficulties perceived by men | 2 classes  With time in mixture  BIC 1226.2 | Class 1 (71.8%) M = 7.52, SE = 0.72, p < .001 with no significant changes over time (post-fu1 p = .741, post-fu2 p = .588)  Class 2(28.2%) M = 19.34, SE = 1.38, p < .001 with significant decreases over time from post to fu1 [B = -5.94, SE = 1.58, p < .001] and from post to fu2 [B = -4.08, SE = 1.50, p = .006]  Class 2 men are significantly less likely to be attributed to Class 1 [OR = 0.98, SE = 0.35, p = .005] | G(4) = 17.8, p = .001  Cramer's V: .53 |

**Table S2**

*Number of Observations (n), Empirical Means (M), and Standard Deviations (SD) of Target Variables at Each Measurement Time for Women and Men*

|  | Post | | | | | | |  | Follow-up 1 | | | | | | |  | Follow-up 2 | | | | | | |
| --- | --- | --- | --- | --- | --- | --- | --- | --- | --- | --- | --- | --- | --- | --- | --- | --- | --- | --- | --- | --- | --- | --- | --- |
|  | Women | | |  | Men | | |  | Women | | |  | Men | | |  | Women | | |  | Men | | |
|  | *n* | *M* | *SD* |  | *n* | *M* | *SD* |  | *n* | *M* | *SD* |  | *n* | *M* | *SD* |  | *n* | *M* | *SD* |  | *n* | *M* | *SD* |
| OQ | 87 | 13.72 | 6.37 |  | 88 | 13.69 | 6.44 |  | 76 | 13.53 | 6.96 |  | 74 | 14.34 | 6.27 |  | 72 | 12.46 | 5.35 |  | 70 | 12.76 | 5.83 |
| PHQ | 87 | 6.59 | 4.45 |  | 88 | 5.73 | 4.75 |  | 76 | 5.86 | 4.34 |  | 74 | 6.26 | 5.40 |  | 72 | 5.55 | 4.30 |  | 70 | 5.26 | 4.13 |
| DAS | 86 | 93.41 | 20.79 |  | 84 | 100.42 | 16.71 |  | 71 | 94.79 | 21.19 |  | 70 | 99.25 | 17.54 |  | 66 | 95.47 | 21.85 |  | 65 | 101.65 | 21.11 |
| PAM | 87 | 78.23 | 12.48 |  | 87 | 82.46 | 11.52 |  | 76 | 77.64 | 13.86 |  | 74 | 81.68 | 11.92 |  | 72 | 77.94 | 14.09 |  | 70 | 81.07 | 12.99 |
| CI-PA | 87 | 1.07 | 0.6 |  | 86 | 1.01 | 0.63 |  | 76 | 1.12 | 0.66 |  | 74 | 1.07 | 0.64 |  | 72 | 1.02 | 0.68 |  | 70 | 1.08 | 0.65 |
| SDQ | 70 | 11.19 | 7.94 |  | 66 | 10.74 | 6.80 |  | 66 | 8.98 | 5.21 |  | 65 | 9.04 | 4.92 |  | 67 | 8.90 | 6.58 |  | 67 | 9.27 | 5.55 |
| *Note.* OQ = Outcome Questionnaire, PHQ = Patient Health Questionnaire, DAS = Dyadic Adjustment Scale, PAM = Parenting Alliance  Measure, CI-PA = Coparenting Inventory for Parents and Adolescents, SDQ = Strengths and Difficulties Questionnaire. | | | | | | | | | | | | | | | | | | | | | | | |

**Table S3**

*The Full Model for Individual Symptomatology (OQ) as the Outcome Variable*

|  | **OQ** | | |
| --- | --- | --- | --- |
| *Predictors* | *Estimates* | *CI* | *p* |
| G0 * M | 13.49 | 11.65 – 15.33 | **<0.001** |
| G0 * W | 13.48 | 11.65 – 15.30 | **<0.001** |
| G1 * M | 14.51 | 12.79 – 16.22 | **<0.001** |
| G1 * W | 14.07 | 12.34 – 15.79 | **<0.001** |
| G0 * M * time | -0.49 | -1.31 – 0.33 | 0.245 |
| G0 * W * time | -0.68 | -1.49 – 0.14 | 0.108 |
| G1 * M * time | -0.10 | -0.89 – 0.68 | 0.799 |
| G1 * W * time | -0.12 | -0.89 – 0.65 | 0.770 |
| **Random Effects** | | | |
| σ^2^ | 12.37 | | |
| τ_00_ _Couple_ | 12.40 | | |
| τ_11_ _Couple.M_ | 22.10 | | |
| τ_11_ _Couple.W_ | 21.89 | | |
| ρ_01_ | -0.26 | | |
|  | -0.26 | | |
| ICC | 0.50 | | |
| N _Couple_ | 88 | | |
| Observations | 467 | | |
| Marginal R^2^ / Conditional R^2^ | 0.021 / 0.511 | | |

*Note.* G0 = BST-as-usual, G1 = IBSI, W = woman, M = man.

**Table S4**

*The Full Model for Depression (PHQ-9) as the Outcome Variable*

|  | **PHQ** | | |
| --- | --- | --- | --- |
| *Predictors* | *Estimates* | *CI* | *p* |
| G0 * M | 5.43 | 4.00 – 6.86 | **<0.001** |
| G0 * W | 6.32 | 5.03 – 7.61 | **<0.001** |
| G1 * M | 6.46 | 5.13 – 7.80 | **<0.001** |
| G1 * W | 6.60 | 5.38 – 7.82 | **<0.001** |
| G0 * M * time | 0.03 | -0.63 – 0.68 | 0.939 |
| G0 * W * time | -0.68 | -1.34 – -0.03 | **0.042** |
| G1 * M * time | -0.10 | -0.73 – 0.53 | 0.756 |
| G1 * W * time | -0.09 | -0.70 – 0.53 | 0.787 |
| **Random Effects** | | | |
| σ^2^ | 7.85 | | |
| τ_00_ _Couple_ | 7.30 | | |
| τ_11_ _Couple.M_ | 13.85 | | |
| τ_11_ _Couple.W_ | 11.92 | | |
| ρ_01_ | -0.30 | | |
|  | -0.43 | | |
| ICC | 0.48 | | |
| N _Couple_ | 88 | | |
| Observations | 467 | | |
| Marginal R^2^ / Conditional R^2^ | 0.018 / 0.491 | | |

*Note.* G0 = BST-as-usual, G1 = IBSI, W = woman, M = man.

**Table S5**

*The Full Model for Relationship Satisfaction (DAS) as the Outcome Variable*

|  | **DAS** | | |
| --- | --- | --- | --- |
| *Predictors* | *Estimates* | *CI* | *p* |
| G0 * M | 99.52 | 93.89 – 105.15 | **<0.001** |
| G0 * W | 93.01 | 86.63 – 99.40 | **<0.001** |
| G1 * M | 99.52 | 94.15 – 104.88 | **<0.001** |
| G1 * W | 93.95 | 87.80 – 100.10 | **<0.001** |
| G0 * M * time | -0.75 | -3.34 – 1.84 | 0.571 |
| G0 * W * time | -0.92 | -3.50 – 1.65 | 0.484 |
| G1 * M * time | -1.17 | -3.62 – 1.28 | 0.354 |
| G1 * W * time | -1.44 | -3.88 – 1.00 | 0.251 |
| **Random Effects** | | | |
| σ^2^ | 112.21 | | |
| τ_00_ _Couple_ | 145.80 | | |
| τ_11_ _Couple.M_ | 151.62 | | |
| τ_11_ _Couple.W_ | 201.60 | | |
| ρ_01_ | -0.19 | | |
|  | 0.00 | | |
| ICC | 0.57 | | |
| N _Couple_ | 88 | | |
| Observations | 442 | | |
| Marginal R^2^ / Conditional R^2^ | 0.039 / 0.582 | | |

*Note.* G0 = BST-as-usual, G1 = IBSI, W = woman, M = man.

**Table S6**

*The Full Model for Coparenting Support (PAM) as the Outcome Variable*

|  | **PAM** | | |
| --- | --- | --- | --- |
| *Predictors* | *Estimates* | *CI* | *p* |
| G0 * M | 82.68 | 79.09 – 86.27 | **<0.001** |
| G0 * W | 78.96 | 74.90 – 83.01 | **<0.001** |
| G1 * M | 82.12 | 78.74 – 85.50 | **<0.001** |
| G1 * W | 77.09 | 73.28 – 80.91 | **<0.001** |
| G0 * M * time | -1.84 | -3.38 – -0.30 | **0.020** |
| G0 * W * time | -1.24 | -2.79 – 0.30 | 0.117 |
| G1 * M * time | -0.56 | -2.04 – 0.92 | 0.459 |
| G1 * W * time | -0.31 | -1.76 – 1.15 | 0.681 |
| **Random Effects** | | | |
| σ^2^ | 43.43 | | |
| τ_00_ _Couple_ | 54.18 | | |
| τ_11_ _Couple.M_ | 62.57 | | |
| τ_11_ _Couple.W_ | 81.63 | | |
| ρ_01_ | -0.16 | | |
|  | 0.00 | | |
| ICC | 0.56 | | |
| N _Couple_ | 88 | | |
| Observations | 466 | | |
| Marginal R^2^ / Conditional R^2^ | 0.050 / 0.577 | | |

*Note.* G0 = BST-as-usual, G1 = IBSI, W = woman, M = man.

**Table S7**

*The Full Model for Coparenting Conflict (CIPA) as the Outcome Variable*

|  | **CIPA** | | |
| --- | --- | --- | --- |
| *Predictors* | *Estimates* | *CI* | *p* |
| G0 * M | 1.03 | 0.84 – 1.23 | **<0.001** |
| G0 * W | 1.07 | 0.88 – 1.27 | **<0.001** |
| G1 * M | 0.99 | 0.81 – 1.17 | **<0.001** |
| G1 * W | 1.10 | 0.92 – 1.29 | **<0.001** |
| G0 * M * time | 0.05 | -0.02 – 0.13 | 0.151 |
| G0 * W * time | -0.01 | -0.08 – 0.06 | 0.794 |
| G1 * M * time | 0.07 | -0.00 – 0.14 | 0.061 |
| G1 * W * time | 0.02 | -0.05 – 0.08 | 0.655 |
| **Random Effects** | | | |
| σ^2^ | 0.10 | | |
| τ_00_ _Couple_ | 0.12 | | |
| τ_11_ _Couple.M_ | 0.20 | | |
| τ_11_ _Couple.W_ | 0.21 | | |
| ρ_01_ | -0.08 | | |
|  | -0.04 | | |
| ICC | 0.56 | | |
| N _Couple_ | 88 | | |
| Observations | 465 | | |
| Marginal R^2^ / Conditional R^2^ | 0.009 / 0.564 | | |

*Note.* G0 = BST-as-usual, G1 = IBSI, W = woman, M = man.

**Table S8**

*Comparison of Post-test Scores with Those at Follow-up 2 Using Paired Student’s t-tests*

|  | Women | | | | |  | Men | | | | |
| --- | --- | --- | --- | --- | --- | --- | --- | --- | --- | --- | --- |
|  | *M_post_* | *M_fu2_* | *n_paired_* | *t* | *p* |  | *M_post_* | *M_fu2_* | *n_paired_* | *t* | *p* |
| OQ | 13.24 | 12.54 | 67 | 1.02 | .312 |  | 13.26 | 12.65 | 65 | 1.10 | .278 |
| PHQ | 6.24 | 5.62 | 67 | 1.17 | .247 |  | 5.15 | 5.23 | 65 | -0.19 | .851 |
| DAS | 98.32 | 94.89 | 60 | 1.44 | .156 |  | 103.55 | 101.74 | 58 | 0.87 | .390 |
| PAM | 79.70 | 77.76 | 67 | 1.74 | .086 |  | 83.45 | 81.02 | 65 | 1.73 | .088 |
| CI-PA | 1.03 | 1.03 | 67 | .000 | .998 |  | 0.96 | 1.09 | 65 | -2.00 | **.049** |
| SDQ | 10.65 | 8.61 | 51 | 2.55 | **.014** |  | 10.64 | 8.8 | 50 | 2.36 | **.022** |

*Note.* Means were computed using only paired observations. Statistical tests significant at the 5% threshold are highlighted in bold. OQ = Outcome Questionnaire, PHQ = Patient Health Questionnaire, DAS = Dyadic Adjustment Scale, PAM = Parenting Alliance Measure, CI-PA = Coparenting Inventory for Parents and Adolescents, SDQ = Strengths and Difficulties Questionnaire.

**Table S9**

***Linear Mixed Model Parameters Describing the Evolution of Outcome Variables for Women and Men***

|  |  |  | *Women* |  |  |
| --- | --- | --- | --- | --- | --- |
|  | *M_post_* | *M_fu1 -_ M_post_* |  | *M_fu2 -_ M_post_* |  |
| OQ | 13.75 | 0.09 | *t*(143.22) = -0.14, *p* = .888 | -0.86 | *t*(143.73) = -1.30, *p* = .195 |
| PHQ | 6.57 | -0.59 | *t*(143.98) = -1.25, *p* = .214 | -0.73 | *t*(144.59) = -1.52, *p* = .131 |
| DAS | 93.28 | -1.21 | *t*(126.30) = -0.59, *p* = .553 | -2.49 | *t*(126.72) = -1.20, *p* = .234 |
| PAM | 78.14 | -1.44 | *t*(138.00) = -1.32, *p* = .191 | -1.50 | *t*(138.28) = -1.35, *p* = .178 |
| CI-PA | 1.09 | 0.10 | *t*(137.86) = -2.00, *p* = **.048** | -0.01 | *t*(138.08) = -0.30, *p* = .769 |
| SDQ | 11.31 | -2.41 | *t*(119.73) = -2.88, *p* = **.005** | -2.09 | *t*(123.26) = -2.47, *p* = **.015** |

|  |  |  | *Men* |  |  |
| --- | --- | --- | --- | --- | --- |
|  | *M_post_* | *M_fu1 -_ M_post_* |  | *M_fu2 -_ M_post_* |  |
| OQ | 13.66 | 0.82 | *t*(137.80) = -1.37, *p* = .173 | -0.67 | *t*(138.17) = -1.09, *p* = .277 |
| PHQ | 5.70 | 0.77 | *t*(136.02) = -1.50, *p* = .136 | -0.09 | *t*(136.51) = -0.18, *p* = .862 |
| DAS | 100.66 | -2.77 | *t*(128.62) = -1.57, *p* = .119 | -1.06 | *t*(129.09) = -0.59, *p* = .556 |
| PAM | 82.53 | -1.41 | *t*(137.92) = -1.22, *p* = .225 | -2.18 | *t*(138.30) = -1.86, *p* = .065 |
| CI-PA | 0.99 | 0.08 | *t*(136.34) = 1.52, *p* = .130 | 0.13 | *t*(136.63) = -2.34, *p* = **.021** |
| SDQ | 10.75 | -1.89 | *t*(118.51) = -2.57, *p* = **.011** | -1.40 | *t*(120.62) = -1.91, *p* = .059 |

*Note. M_post_* **is the model intercept;** *M_fu1 -_ M_post_* **represents the parameter associated with the first contrast, corresponding to the change between follow-up 1 and the post-test;** *M_fu2 -_ M_post_* **represents the parameter associated with the second contrast, corresponding to the change between follow-up 2 and the post-test.** Statistical tests significant at the 5% threshold are highlighted in bold. OQ = Outcome Questionnaire, PHQ = Patient Health Questionnaire, DAS = Dyadic Adjustment Scale, PAM = Parenting Alliance Measure, CI-PA = Coparenting Inventory for Parents and Adolescents, SDQ = Strengths and Difficulties Questionnaire.

**Figure S1**

*Illustration of the Different Groups for the Individual Symptomatology (OQ) Outcome Variable*

**Figure S2**

*Illustration of the Different Groups for the Depression (PHQ-9) Outcome Variable*

**Figure S3**

*Illustration of the Different Groups for the Relationship Satisfaction (DAS) Outcome Variable*

**Figure S4**

*Illustration of the Different Groups for the Coparenting Support (PAM) Outcome Variable*

**Figure S5**

*Illustration of the Different Groups for the Coparenting Conflict (CIPA) Outcome Variable*

**Figure S6**

*Illustration of the Different Groups for the Child’s Strength and Difficulties (SDQ) Outcome Variable*
